# A novel method for the determination of isoxazoline derivatives in plasma by ultra-high performance liquid chromatography–tandem mass spectrometry: validation and applicability to screening tests

**DOI:** 10.2478/jvetres-2025-0050

**Published:** 2025-09-24

**Authors:** Paulina Markowska-Buńka, Jerzy J. Jaroszewski, Bartosz Rasiński, Hubert Ziółkowski

**Affiliations:** 1 Department of Pharmacology and Toxicology, Faculty of Veterinary Medicine, University of Warmia and Mazury, 10-719 Olsztyn, Poland; 2 Waters Sp. z o.o., 01-531 Warsaw, Poland

**Keywords:** afoxolaner, fluralaner, lotilaner, sarolaner, UPLC-MS/MS

## Abstract

**Introduction:**

Isoxazoline derivatives such as fluralaner, sarolaner, lotilaner, and afoxolaner are a new class of insecticide and acaricide compounds. These compounds are characterised by rapid action and high efficacy over a period of up to several weeks. A method for identifying all four isoxazoline derivatives in animal and human plasma has not been proposed to date. Therefore, the aim of this study was to develop and validate a novel analytical method for the determination of fluralaner, sarolaner, lotilaner, and afoxolaner in laying hen plasma and to revalidate the proposed method in human, canine and feline plasma.

**Material and Methods:**

The plasma concentrations of isoxazoline derivatives were determined by ultra-high performance liquid chromatography–tandem mass spectrometry. Analytical samples were prepared by precipitating proteins with a mixture of 96% acetonitrile and 4% ammonium hydroxide (v/v). Chromatographic separation was achieved on a chromatographic column (1.8 μm 2.1 × 100 mm) using 0.1% formic acid in water and 0.1% formic acid in acetonitrile with gradient elution.

**Results:**

The results indicate that the described method is replicable, linear (r^2^ = 0.99), precise (1.66% to 14.97%), accurate (1.19% to 11.67%), selective and sensitive (limit of quantitation = 1 ng/mL). Depending on the studied compound and animal species, total recovery reached 85–99%, and the matrix effect did not exceed 15% in any of the analyses.

**Conclusion:**

The proposed method is simple, effective, inexpensive and rapid because it requires only a single-step sample preparation protocol.

## Introduction

The diseases, some of which are parasitic, transmitted by arthropods such as ticks or fleas pose a serious health threat to pets and farm animals. Antiparasitic drugs are one of the largest groups of veterinary medications (9). Antiparasitic compounds include the isoxazoline derivatives that have been developed in the last 25 years. These include fluralaner, sarolaner, lotilaner, afoxolaner and esafoxolaner. A single oral dose of isoxazoline derivatives provides effective protection against arthropods for up to 12 weeks. These drugs have a rapid onset of action, which reduces the risk of disease transmission by ectoparasites (18, 19). In recent years, single and combined isoxazoline products have been approved by the American Food and Drug Administration and the European Medicines Agency, and these drugs are widely used to treat ectoparasite infections. Fluralaner, sarolaner and lotilaner have been approved for use in dogs and cats, afoxolaner has been approved for dogs (19), esafoxolaner for cats (15), fluralaner for hens (7, 11, 14) and lotilaner for humans (13). Despite the growing popularity of isoxazoline derivatives, very few studies have investigated their presence in biological matrices using liquid chromatography coupled to tandem mass spectrometry (LC-MS/MS) (1, 2, 5, 10, 16, 17). Most of the described analytical methods support the determination of only one isoxazoline derivative in a given biological matrix (2, 5, 10, 16). These methods are also characterised by low repeatability due to incomplete information about equipment settings and extraction techniques. Therefore, the first aim of this study was to develop a sensitive, simple, inexpensive and rapid method for analysing isoxazoline compounds. The second aim was to revalidate the proposed method for use in human plasma and the plasma of three animal species, as well as in the first screening test of plasma obtained from a small animal clinic. Ultra-high performance (UP) LC-MS/MS was the technique selected to achieve the aims.

## Material and Methods

### Screening tests

Blood samples for the analyses were obtained from 20 randomly selected, clinically healthy dogs (7 German shepherds and 2 golden retrievers aged 2–10 years; and 3 Yorkshire terriers, 3 schnauzers, 2 Siberian huskies, 2 beagles and 1 Labrador retriever, all aged 2–5 years) and from 10 randomly selected, clinically healthy cats (5 European and 5 Persian, all aged 3–10 years). Blood was not sampled specifically for the study, but was drawn during routine veterinary examinations with the owners’ consent. Blood that was not used for routine health examinations was donated to the researchers by courtesy of the owners and veterinary physicians. This method of acquiring biological material for research did not require the approval of the Ethics Committee. Blood was drawn from the brachial vein into heparinised sterile tubes (BD Biosciences, San Jose, CA, USA). Plasma was separated by centrifugation at 1,650 × *g* for 10 min at 4°C and was stored at -70°C until analysis.

### Sample preparation

Analyte-free plasma

obtained from clinically healthy animals and humans was thawed at room temperature and shaken in a vortex mixer at 1,000 rpm for 5 s. Plasma samples of 100 μL each were transferred to clean 5 mL polyethylene tubes and combined with 10 μL of fluralaner, sarolaner, lotilaner or afoxolaner and 10 μL of the fluralaner or sarolaner internal standard solution. The samples were shaken at 1,000 rpm for 5 s. Next, 450 μL of acetonitrile (ACN) : NH3 (96 : 4; v/v) was added for protein precipitation, and the samples were shaken at 3,000 rpm for 30 s. After centrifugation at 20,000 × *g* for 10 min at 4°C, 150 μL of the supernatant was passed through a 0.22 μm nylon syringe filter with a diameter of 13 mm (Waters, Milford, MA, USA) into chromatographic total recovery vials and injected into the LC-MS/MS system.

### Chemicals and reagents

The reagents for LC-MS/MS, including ACN, NH3, methanol, water and formic acid (all LC/MS grade), were purchased from Sigma-Aldrich (Darmstadt, Germany). Analytical standards of fluralaner, sarolaner, lotilaner and afoxolaner (purity ≥98%) were supplied by HPC Standards GmbH (Borsdorf, Germany). Nitrogen for the LC-MS/MS system was supplied by a NitroGen N110R nitrogen generator (Peak Scientific, Inchanian, UK) and argon was supplied by Eurogaz-Bombi (Olsztyn, Poland).

### Instruments and conditions

The analyses were conducted using an Acquity UPLC system coupled to a XEVO TQ-XS triple quadrupole tandem mass spectrometer (Waters). Chromatographic separation was performed on an Acquity HSS (high-strength silica) T3 column (1.8 μm, 2.1 × 100 mm; Waters), and the mobile phase consisted of phase A (0.1% formic acid in ACN) and phase B (0.1% formic acid in water). The gradient elution programme of the mobile phase was as follows: 35% A in 0–2.6 min, 28% A in 2.7–2.9 min, 0% A in 3.0–3.4 min and 35% A from 3.50 min (Table 1). Each analysis lasted 5.5 min at a flow rate of 0.35 mL/min, including 3.5 min for chromatographic separation and 2 min for column re-equilibration. The injection volume was 1 μL, autosampler temperature was maintained at 15°C, and column temperature was maintained at 35°C. The MS/MS system was operated in electrospray positive ionisation mode (Table 1).

**Table 1.** Selected parameters of ultra-high performance liquid chromatography–tandem mass spectrometry (MS/MS) for the determination of isoxazoline derivatives in plasma

MS/MS parameters		Compound			
		Fluralaner	Sarolaner	Lotilaner	Afoxolaner
Precursor ions (m/z)		556.06	581.03	595.98	625.73
Product ions (m/z)	Quantification ions	457.0	457.0	439.75	469.89
	Confirmation ions	400.0	415.9	166.03	195.97
Collision energy (eV)	Quantification ions	18	25	30	26
	Confirmation ions	12	20	35	48
Retention time (min)		2.55	2.55	3.33	3.18
Capillary voltage (kV)			3.0		
Cone voltage (V)			20		
Desolvation gas			Nitrogen		
Desolvation gas temperature (°C)			600		
Desolvation gas flow (L/h)			1000		
Collision gas flow (mL/min)			0.2		
Collision gas			Argon		
Source temperature (°C)			120		
Electrospray mode			Positive		
Dwell (s)			0.1		

Time (min)	Flow rate (mL/min)	Mobile phase (%)			Curve
		A	B		
0.00	0.35	35.0	65.0		Initial
2.70	0.35	28.0	72.0		6
3.00	0.35	0.0	100.0		6
3.50	0.35	35.0	65.0		11
Mobile phase		0.1% formic acid in acetonitrile	0.1% formic acid in water		
Column	ACQUITY HSS T3, 1.8 μm 2.1 × 100 mm				

### Stock solution, calibration curves and quality control samples

Stock solutions of fluralaner, sarolaner, lotilaner and afoxolaner were prepared by dissolving 0.5 mg of analytical standards in 5 mL of methanol. Sarolaner was the internal standard for fluralaner, lotilaner and afoxolaner, whereas fluralaner was the internal standard for sarolaner. The stock solutions were prepared in 5 mL volumetric borosilicate flasks (Duran Group, Mainz, Germany). A series of working standard solutions (all for the calibration curve) were prepared for validation by diluting the stock solutions with methanol. These solutions were prepared in 5 mL volumetric borosilicate flasks by diluting stock solutions in methanol at the following concentrations: 0.025, 0.125, 0.25, 0.625, 1.25, 2.5, 6.25, 12.5, 18.75 and 37.5 μg/mL. All solutions were stored at 4°C in the refrigerator and were brought to room temperature before use. Five calibration points solutions were additionally used to prepare quality control (QC) points: high QC (HQC) – 37.5 μg/mL, medium QC (MQC) – 25 μg/mL, intermediate QC (IQC) – 2.5 μg/mL (50% of the calibration curve), low QC (LQC) – 0.125 μg/mL and the lower limit of quantification (LLOQ) – 0.025 μg/mL.

### Development and validation of the analytical method

The analytical method was validated as described by Kruve *et al*. (3, 4). The following parameters were determined during the validation procedure: linearity, accuracy, precision (repeatability/intra-day precision and intermediate precision/inter-day precision), limit of detection (LOD), LLOQ, selectivity, recovery, matrix effect, carry-over and stability (freeze– thaw stability, autosampler stability, working standard, stock stability and stability of sample processing temperature) (Table 2). All validation procedures were conducted in plasma sampled from clinically healthy laying hens. The method was then revalidated for selectivity, the matrix effect and total recovery based on the procedure described by Markowska *et al*. (6).

### Linearity

Ten calibration points were developed for the linearity test (0.001, 0.005, 0.01, 0.025, 0.05, 0.1, 0.25, 0.5, 0.75 and 1.5 μg/mL) for fluralaner, sarolaner, lotilaner and afoxolaner. The calibration points were prepared five times at daily intervals. Each curve was analysed twice, and each analysis was preceded by an analysis of a sample with no analytes (blank sample) and a sample containing only the internal standard (zero sample). The concentrations of isoxazoline derivatives in the plasma of laying hens were subjected to a linear regression analysis using the following regression equation: y = ax + b (Table 2).

**Table 2.** Calculation methods and acceptance criteria for parameters for validation of an ultra-high performance liquid chromatography–tandem mass spectrometry method for the determination of isoxazoline derivatives in plasma

	Parameter	Acceptance criteria
Linearity	Calibration points	Back-calculated concentrations should be within ± 15% of the nominal concentration, and at least 75% of the calibration points, but no fewer than 6, must fulfil this criterion
	Coefficient of determination (r^2^)	≥ 0.99
	Relative residuals (Yi)	$\left|\frac{y_{i}-{\hat{y}}_{i}}{{\hat{y}}_{i}}\right|\times 100\%\leq 20\%$
	SD of relative residuals (S_Yi_)	$\sqrt{\frac{\sum {\left(Y_{i}-\bar{Y}\right)}^{2}}{n-2}}\leq 0.1$
Stability	Stock and working standard	$\frac{S_{t}}{S_{0}}\times 1100\%=within\pm 15\%ofS_{0}$
	Autosampler	
	Freeze and thaw	
	Sample processing temperature	
Precision (RSD or CV)		$\frac{SD}{C_{mean\;}}\times 100\%=\;within\;\pm 15\%\;of\;the\;nominal\;concentration$
Accuracy (deviation) (for at least 5 points per group/day)		$\frac{\left|\left(C_{t}-C_{n}\right)\right|}{C_{n}}\times 100\%=\;within\;\pm 15\%\;of\;the\;\;nominal\;concentration$
LOD		3 × *SD_C__fortified_* where *S*/*N* ≥ 3: 1
LLOQ with accuracy and precision		6 × *C_fortified_* where *S*/*N* ≥ 10: 1
Matrix effect		$100-\left(\frac{X_{i}}{X}\times 100\%\right)=\pm 15\%\;relative\;to\;the\;sample\;without\;the\;matrix$
Total recovery		$\frac{X_{z}}{X_{i}}\times 100\%$
Selectivity/Specificity		No endogenous peaks in the retention time of the analyte
Carry-over		Area of carry-over peaks: ≤ 20% of LLOQ and 5% of IS area

1 y_i_ – experimental signal; ŷ_i_ – calculated signal; SD – standard deviation; Y_i_ – relative residual; Ȳ – mean value of relative residuals; S_t_ – peak area when the analysis was paused for time *t*; S_0_ – initial peak area without additional pauses during the analysis (freshly prepared standards); RSD – relative standard deviation; CV – coefficient of variation; C_mean_ – mean concentration (ng/mL); C_t_ – individually calculated concentration (ng/mL); C_n_ – nominal concentration (ng/mL); LOD – limit of detection; SD_Cfortified_ – standard deviation calculated from fortified samples with the lowest acceptable concentration; S/N – signal-to-noise; LLOQ – lower limit of quantification; C_fortified_ – minimal fortified concentration which meets the requirements; X_i_ – peak area of the analyte added to the matrix after extraction; X – peak area of the analyte in the final solvent; X_z_ – peak area of the analyte added to the matrix before extraction

### Precision and accuracy

Precision and accuracy were determined during daily analyses and between analytical dates. The QC points were prepared six times over a period of three days. Five calibration points were used to prepare QC as follows: HQC 37.5 μg/mL, MQC 25 μg/mL, IQC 1.5 μg/mL (50% of the calibration curve), LQC 0.125 μg/mL and LLOQ 0.025 μg/mL. It was specified for precision and accuracy for all QC points that they should not exceed ±15% of the nominal concentration and ±20% of the LLOQ (Table 2).

### Stability

The stability of the studied isoxazoline compounds was estimated at all stages of sample storage, preparation and analysis. Stability tests were conducted after 3 h of storage at room temperature; after 24 h of storage in an autosampler at 4°C; after 24, 48 and 72 h of freezing/thawing at –80°C; and after 20 d of storage. Stability tests were prepared in six replicates for each QC point. Each QC sample was analysed relative to a freshly prepared calibration curve. The mean concentrations were within ±15% of the nominal concentration (Table 2).

### Selectivity

**Fig 1.**
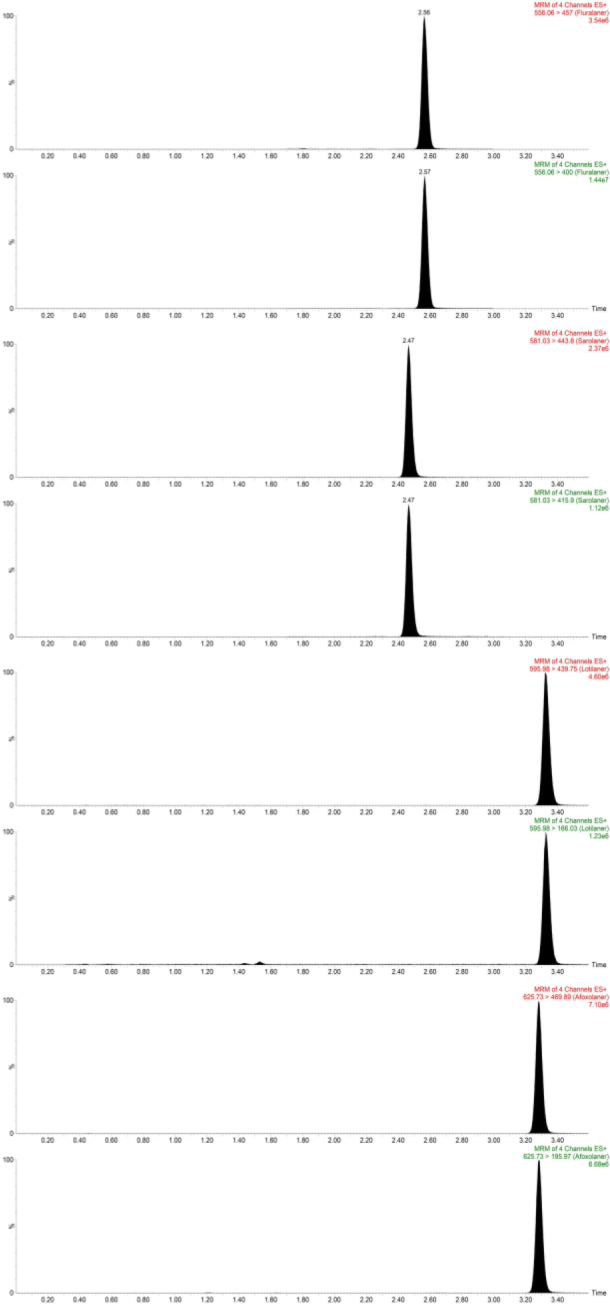
Liquid chromatography–tandem mass spectrometry traces of separation of four isoxazoline derivatives from plasma (fluralaner, sarolaner, lotilaner and afoxolaner)

The selectivity of the method was validated based on the presence or absence of interferences and by comparing the chromatograms of six blank plasma samples from laying hens, dogs, cats and humans, and blank serum samples spiked with the standard. The absence of interfering components was accepted when the response was 20% or less of the LLOQ for fluralaner, sarolaner, lotilaner and afoxolaner, and 5% or less for the internal standard (Table 2, Fig 1.).

### Recovery

Extraction recovery was assessed in six replicates of each QC point, where the analytical standards were added before and after extraction. The peak area of the analyte and the internal standard added after the extraction was indicative of 100% recovery (Table 2).

### Matrix effect

To evaluate the matrix effect, the analytical standards and the internal standard were added to plasma samples after extraction. Next, the signal from each compound was compared to the signal produced by fluralaner, sarolaner, lotilaner, afoxolaner and the internal standard that was added to a mixture of water (replacing plasma) and ACN : NH3 and was also prepared in six replicates for each QC point. It was specified that the increase or decrease in the extraction rate should not exceed ±15% (Table 2).

### Carry-over

This test was conducted to eliminate any carry-over of isoxazoline derivatives between the samples in any part of the chromatography system (injector, column, mobile phase, *etc*.). The carry-over effect was assessed in six replicates by injecting a single blank sample after a HQC sample, and it was regarded as acceptable at ≤20% of the LLOQ (Table 2).

## Results

The mass spectrometer was operated in electrospray positive ionization (ESI+) mode. The spectra were traced in multiple-reaction-monitoring mode at 556.06 m/z to 400.0 and 457.0 for fluralaner, 581.03 m/z to 457.0 and 415.9 for sarolaner, 595.98 m/z to 439.75 and 166.03 for lotilaner and 625.73 to 469.89 and 195.97 for afoxolaner (Table 1).

The method developed in this study was fully validated in laying hen plasma. Additionally a validation outcome emerged from the results for total recovery and the matrix effect from samples of canine, feline and human plasma; these results are shown in Table 3. The results of the validation protocol met all acceptance criteria (6) (Table 2). The values of r and r^2^ in the linear regression analysis of the calibration curve were above 0.99 (Tables 4–7), and the entire calibration range was 0.001–1.5 μg/mL. Accuracy and precision were estimated for all QCs and the LLOQ, both for individual samples and for the six samples prepared over a period of three days. Intra-day precision was 1.79% to 14.97%, and accuracy was 1.33% to 11.67% for all QC and LLOQ (Tables 4–7).

**Table 3.** Validation results for total recovery by ultra-high performance liquid chromatography–tandem mass spectrometry for the determination of isoxazoline derivatives in plasma from clinically healthy dogs, cats and humans and validation results for matrix effect

Validation test		Dog				Cat				Human			
		Fluralaner	Sarolaner	Lotilaner	Afoxolaner	Fluralaner	Sarolaner	Lotilaner	Afoxolaner	Fluralaner	Sarolaner	Lotilaner	Afoxolaner
Total recovery (%)	Mean	13.10	13.90	10.10	13.90	11.15	14.10	9.80	13.6	12.10	13.00	11.93	10.70
	SD	2.10	1.80	1.18	2.00	1.43	1.12	2.50	2.20	2.90	0.80	0.90	1.10
Matrix effect (%)	Mean	8.90	13.90	13.30	12.10	12.20	9.80	9.50	11.90	13.60	13.70	8.93	9.60
	SD	1.54	1.89	1.04	2.03	2.12	2.34	2.12	1.07	1.14	2.01	1.08	1.71

1 SD – standard deviation

**Table 4.** Validation results of an ultra-high performance liquid chromatography–tandem mass spectrometry method for the determination of fluralaner in clinically healthy laying hen plasma

Linearity	_r_2		I 0.99	II 0.99	III 0.99	IV 0.99	V 0.99	Mean 0.99
Precision (%) and accuracy (%)	Intra-day, n = 6; 3 replications			LLOQ	LQC	IQC	MQC	HQC
		Precision	I	8.94	5.59	1.94	3.89	1.66
		Accuracy		6.67	4.33	1.62	2.64	1.29
		Precision	II	4.15	6.81	2.34	7.81	2.47
		Accuracy		1.67	5.33	1.90	4.65	1,99
	Inter-day, n = 18	Precision	III	7.66	9.38	5.54	3.25	6.16
		Accuracy		5.00	7.33	4.60	6.01	4,87
		Precision		7.23	2.19	1.75	3.03	3.16
		Accuracy		5.70	1.67	4.91	2.90	2.60
			Concentration (ng/mL)			S/N		
LLOQ and LOD	LLOQ overall mean, n = 18		1.00			90.00		
	LLOQ overall SD, n = 18		0.14			5.91		
	LOD overall mean, n = 18		0.5			6.22		
	LOD overall SD, n = 18		0.33			3.91		
Total recovery	Sample		Total recovery (%)					
	Mean	1 ng/mL	91.19					
		1,500 ng/mL	85.39					
	SD	1 ng/mL	12.73					
		1,500 ng/mL	5.94					
			Matrix effect (%)					
Matrix effect	Mean	1 ng/mL	10.15					
		1,500 ng/mL	14.81					
	SD	1 ng/mL	8.22					
		1,500 ng/mL	2.94					
Stability tests results								
Stability	Period (h)		Changes in the concentration of QCs (%)					
			LQC	IQC		MQC		HQC
	24		13.25	3.07		4.96		1.16
Working standard, 2°C	48		8.92	14.58		12.01		14.28
	120		13.25	10.90		9.02		12.24
	168		14.31	13.95		11.15		11.10
Autosampler, 4°C	24		7.01	9.80		14.48		7.72
	24		14.71	13.91		5.30		2.77
Freeze and thaw, –75°C	48		14.07	14.93		12.60		4.65
	72		12.33	14.39		13.98		13.49
	480		14.49	14.77		10.30		8.52
Sample processing, 21°C	3		14.04	9.23		4.01		3.73

1 I–V – replicate number; LLOQ – lower limit of quantification; LQC – low quality control; IQC – intermediate quality control; MQC – medium quality control; HQC – high quality control; LOD – limit of detection; SD – standard deviation

**Table 5.** Validation results of an ultra-high performance liquid chromatography–tandem mass spectrometry method for the determination of sarolaner in clinically healthy laying hen plasma

Linearity	_r_2		I	II	III	IV	V	Mean
			0.99	0.99	0.99	0.99	0.99	0.99
Precision (%) and accuracy (%)	Intra-day, n = 6; 3 replications			LLOQ	LQC	IQC	MQC	HQC
		Precision	I	9.67	6.07	1.67	2.34	2,04
		Accuracy		5.00	4.67	1.35	3.86	1,36
		Precision	II	14.48	3.87	3.95	4.22	7,49
		Accuracy		11.67	3.00	2.67	4.66	5,16
	Inter-day, n = 18	Precision	III	10.95	1.79	2.08	6.71	5,10
		Accuracy		6.67	1.33	1.65	7.08	3,33
		Precision		11.70	3.91	2.57	6.02	4.88
		Accuracy		7.78	3.00	1.89	3.33	3.28
			Concentration (ng/mL)				S/N	
LLOQ and LOD	LLOQ overall mean, n = 18		1.00				91.48	
	LLOQ overall SD, n = 18		0.12				4.74	
	LOD overall mean, n = 18		0.5				4.01	
	LOD overall SD, n = 18		0.36				0.02	
	Sample		Total recovery (%)					
Total recovery	Mean	1 ng/mL	99.78					
		1,500 ng/mL	96.21					
	SD	1 ng/mL	14.09					
		1,500 ng/mL	7.47					
			Matrix effect (%)					
Matrix effect	Mean	1 ng/mL	14.40					
		1,500 ng/mL	6.97					
	SD	1 ng/mL	8.09					
		1,500 ng/mL	7.00					
Stability test results								
Stability	Period (h)		Changes in the concentration of QCs (%)					
			LQC	IQC		MQC		HQC
Working standard, 2°C	24		13.97	12.24		12.35		13.31
	48		12.08	14.38		13.55		14.44
	120		7.60	2.85		11.64		3.20
Autosampler, 4°C	168		5.02	8.81		14.90		12.42
	24		13.51	13.93		2.30		6.93
	24		7.12	14.11		14.92		7.17
Freeze and thaw, –75°C	48		9.58	12.84		4.52		3.91
	72		12.56	13.00		12.40		14.90
	480		7.16	2.30		2.66		10.25
Sample processing, 21°C	3		13.38	14.62		3.21		4.07

1 I–V – replicate number; LLOQ – lower limit of quantification; LQC – low quality control; IQC – intermediate quality control; MQC – medium quality control; HQC – high quality control; LOD – limit of detection; SD – standard deviation

**Table 6.** Validation results of an ultra-high performance liquid chromatography–tandem mass spectrometry method for the determination of lotilaner in clinically healthy laying hen plasma

Linearity	r^2^		I	II	III	IV	V	Mean
			0.99	0.99	0.99	0.99	0.99	0.99
Precision (%) and accuracy (%)	Intra-day, n = 6; 3 replications			LLOQ	LQC	IQC	MQC	HQC
		Precision	I	14.97	1.79	3.67	4.46	7.82
		Accuracy		11.67	1.33	2.58	6.72	5.60
		Precision	II	7.40	4.10	4.95	5.02	4.61
		Accuracy		5.00	3.29	3.27	7.03	3.76
	Inter-day, n = 18	Precision	III	6.32	2.33	5.99	8.22	1.42
		Accuracy		3.33	1.67	4.03	9.11	1.19
		Precision		9.57	2.74	4.87	5.15	4.61
		Accuracy		6.67	2.10	3.29	4.99	3.52
			Concentration (ng/mL)				S/N	
LLOQ and LOD	LLOQ overall mean, n = 18		1.00				73.23	
	LLOQ overall SD, n = 18		0.09				5.95	
	LOD overall mean, n = 18		0.5				5.55	
	LOD overall SD, n = 18		0.39				2.87	
	Sample		Total recovery (%)					
Total recovery	Mean	1 ng/mL	95.50					
		1,500 ng/mL	93.16					
	SD	1 ng/mL	14.76					
		1,500 ng/mL	5.07					
			Matrix effect (%)					
Matrix effect	Mean	1 ng/mL	14.10					
		1,500 ng/mL	7.09					
	SD	1 ng/mL	5.98					
		1,500 ng/mL	4.51					
Stability test results								
Stability	Period (h)		Changes in the concentration of QCs (%)					
			LQC	IQC		MQC		HQC
Working standard, 2°C	24		6.32	7.40		10.90		5.91
	48		8.24	13.25		6.07		8.71
	120		9.58	13.60		2.96		11.92
Autosampler, 4°C	168		8.45	14.87		11.29		12.82
	24		13.50	13.99		9.54		14.94
	24		12.30	7.27		12.88		9.35
Freeze and thaw, –75°C	48		8.75	5.60		11.63		12.44
	72		13.03	2.37		3.95		6.73
	480		13.34	11.42		11.26		9.70
Sample processing, 21°C	3		4.21	5.42		10.18		2.61

1 I–V – replicate number; LLOQ – lower limit of quantification; LQC – low quality control; IQC – intermediate quality control; MQC – medium quality control; HQC – high quality control; LOD – limit of detection; SD – standard deviation

**Table 7.** Validation results of an ultra-high performance liquid chromatography–tandem mass spectrometry method for the determination of afoxolaner in clinically healthy laying hen plasma

Linearity	r^2^		I	II	III	IV	V	Mean
			0.99	0.99	0.99	0.99	0.99	0.99
Precision (%) and accuracy (%)	Intra-day, n = 6; 3 repetitions			LLOQ	LQC	IQC	MQC	HQC
		Precision	I	7.66	10.28	3.04	2.47	3.45
		Accuracy		5.00	8.67	2.48	3.22	2.00
		Precision	II	13.22	4.20	4.30	6.66	8.16
		Accuracy		10.00	2.67	3.27	5.54	6.39
	Inter-day, n = 18	Precision	III	9.67	3.10	2.26	5.23	4.52
		Accuracy		5.00	2.67	1.60	8.02	3.65
		Precision		10.18	5.86	3.20	6.07	5.05
		Accuracy		6.67	4.67	2.45	6.01	4.01
			Concentration (ng/mL)				S/N	
LLOQ and LOD	LLOQ overall mean, n = 18		1.00				92.30	
	LLOQ overall SD, n = 18		0.04				4.8	
	LOD overall mean, n = 18		0.5				0.9	
	LOD overall SD, n = 18		0.44				1.11	
	Sample		Total recovery (%)					
Total recovery	Mean	1 ng/mL	85.19					
		1,500 ng/mL	87.05					
	SD	1 ng/mL	14.14					
		1,500 ng/mL	7.88					
			Matrix effect (%)					
Matrix effect	Mean	1 ng/mL	13.77					
		1,500 ng/mL	14.69					
	SD	1 ng/mL	9.56					
		1,500 ng/mL	10.52					
Stability test results								
Stability	Period (h)		Changes in the concentration of QCs (%)					
			LQC	IQC		MQC		HQC
Working standard, 2°C	24		5.74	3.07		4.96		1.16
	48		14.91	14.58		12.01		14.28
	120		9.82	7.82		11.76		14.80
Autosampler, 4°C	168		12.79	3.31		11.28		13.84
	24		13.15	8.10		9.55		14.14
	24		10.00	12.99		6.47		3.44
Freeze and thaw, –75°C	48		14.10	13.78		14.05		12.39
	72		14.84	3.12		13.48		10.89
	480		12.53	10.90		12.66		8.37
Sample processing, 21°C	3		5.66	14.37		13.26		13.68

1 I–V – replicate number; LLOQ – lower limit of quantification; LQC – low quality control; IQC – intermediate quality control; MQC – medium quality control; HQC – high quality control; LOD – limit of detection; SD – standard deviation

Inter-day precision was 1.75% to 11.70%, and accuracy was 1.67% to 7.78% for all QC and LLOQ (Tables 4–7). The LOD was set at 0.5 ng/mL ± 0.38 (with a minimum signal-to-noise ratio of 10 : 1) and the LLOQ was 1.0 ng/mL ± 0.04–0.14 (Tables 4–7). The analysis of the selectivity/specificity of the method revealed no significant peaks during the retention of isoxazoline derivatives in drug-free plasma separated from the blood of clinically healthy animals. No significant carry-over of fluralaner, sarolaner, lotilaner or afoxolaner was observed in analyses involving high concentrations of these analytes. All analytes appeared to be stable after 3 h of storage at sample processing temperature; after 24 h of storage in an autosampler at 4°C; after 24, 48, 72 and 480 h of thawing and freezing cycles; and after 168 h of refrigerated storage at 2°C (Tables 4–7). The mean matrix effect for all species was 11.99%.

The proposed method was successfully applied to analyse the plasma of randomly selected patients of a small animal clinic for the presence of isoxazoline derivatives. The concentrations of isoxazoline compounds were within the analytical range of the method in 15 out of 20 dogs (1.0–434.6 ng/mL) and in 2 out of 10 cats (23.8–233.3 ng/mL) (Table 8).

**Table 8.** Results of screening tests (concentrations in ng/mL)

Species	Breed	Fluralaner	Sarolaner	Lotilaner	Afoxolaner
Dog	German shepherd	-	434.6	-	-
	German shepherd	-	12.1	-	-
	German shepherd	-	-	-	-
	German shepherd	414.3	-	-	-
	German shepherd	-	-	-	1.3
	German Shepherd	-	19.0	-	-
	German shepherd	346.5	-	-	-
	Yorkshire terrier	**-**	-	-	1.0
	Yorkshire terrier	-	-	-	1.0
	Yorkshire terrier	-	-	-	1.0
	Schnauzer	388.3	-	-	-
	Schnauzer	76.7	-	-	-
	Schnauzer	-	-	-	-
	Golden retriever	-	**-**	-	-
	Golden retriever	-	225.0	-	-
	Siberian husky	-	-	-	-
	Siberian husky	-	196.0	-	-
	Beagle	-	-	-	-
	Beagle	-	5.6	-	-
	Labrador retriever	-	100.6	-	-
Cat	European cat	-	-	-	-
	European cat	-	23.8	-	-
	European cat	-	233.3	-	-
	European cat	-	-	-	-
	European cat	-	-	-	-
	Persian cat	-	-	-	-
	Persian cat	-	-	-	-
	Persian cat	-	-	-	-
	Persian cat	-	-	-	-
	Persian cat	-	-	-	-

## Discussion

The present study was undertaken to develop the first ever method for determining four isoxazoline compounds in plasma samples. The proposed method was developed in several steps. The optimal detector parameters were selected in the first step; the optimal chromatographic conditions were selected in the second step; and a quick, effective, easy and inexpensive analyte extraction method was developed in the third step. In the fourth step, the proposed method was validated in laying hen plasma and revalidated in canine, feline and human plasma. The validated method was successfully used in a screening study to determine the concentrations of isoxazoline derivatives in dogs and cats that were randomly selected in a private veterinary clinic.

The molecular weight of isoxazoline derivatives was 556.29 g/mol for fluralaner, 581.36 g/mol for sarolaner, 596.76 g/mol for lotilaner and 625.87 g/mol for afoxolaner. These values were used to determine the parent ions of the analysed molecules on the assumption that each substance was ionised only once (m/z = 1/1). The optimal chromatographic conditions were selected in the following stage of the study. First, the column was selected based on the properties of isoxazoline derivatives which are lipophilic compounds (XLogP3 of 5.6 for fluralaner, 3.4 for sarolaner, 6.6 for lotilaner and 6.7 for afoxolaner). Shoop *et al*. (15) used a Zorbax SB C18 column (2.1 mm × 50 mm, 5 μm) to determine the plasma concentrations of afoxolaner, whereas Toutain *et al*. (16) determined the plasma levels of lotilaner using a Daicel Chiralpak IA-3 column (150 × 4.6 mm) with a much larger particle size than that applied in the current study. A Zorbax Eclipse Plus C18 column (4.6 × 100 mm, 3.5 μm) was used only by Wu *et al*. (17) to measure the concentrations of all isoxazoline compounds, but in their study, tissues rather than plasma were used as the matrix. In contrast, the column used in the present study had a smaller particle size to achieve higher analytical sensitivity. The Acquity HSS T3 column was also chosen because it is compatible with 100% aqueous solvents. A similar mobile phase to that used in the present experiment was applied by Sari *et al*. (8), but they used methanol instead of acetonitrile. Methanol was not used in this study because it leads to a maximal reduction of baseline resolution. Wu *et al*. (17) used a mobile phase composed of 5 mmol/L of aqueous ammonium formate solution and MeOH. In the present study, however, a buffer was not used to avoid crystallisation, which may occur under mass spectrometry conditions due to solvent evaporation and elevated temperatures in the ion source.

Sample preparation is a critical step in analyses of drug residues. An adequate sample preparation protocol removes matrix interference and improves the sensitivity and robustness of the method. The main goal of this study was to develop a quick, easy, short, sensitive, and low-cost method of plasma purification. Protein precipitation is a short procedure that was well suited to the needs of this study. To date, a short and easy method for extracting lotilaner from plasma has only been proposed by Toutain *et al*. (16). In turn, Kilp *et al*. (2) and Sari *et al*. (8) used pure ACN for protein precipitation and determined the plasma levels of fluralaner by solid-phase extraction. Wu *et al*. (17) also used ACN for extraction, followed by a cleanup step involving a PRiME (protein removal in mass spectrometry experiments) HLB (hydrophilic–lipophilic balance) cartridge, and they analysed four isoxazoline compounds (similarly to the present study), but in tissues, not in plasma, which explains the additional cleanup step. For this reason, the method developed by the cited authors is longer, consists of more steps and is more expensive to validate when adopted for tissue analysis because of the high cost of tissue samples.

The method was successfully validated according to Kruve *et al*. (3, 4). The calibration curves showed linearity in a sixfold wider range than other methods (1, 8, 17). The validation confirmed sensitivity several times higher than that described by other authors (1, 17). The validated method demonstrated acceptable inter-day precision and accuracy across all QC levels and LLOQ. In contrast, Elliot *et al*. (1) reported lower precision, with inter-day values exceeding 19.5% and falling out of the ranges of all parameters except LLOQ in our protocol. The developed method demonstrated high analyte stability under all tested conditions, whereas Elliot *et al*. (1) and Wu *et al*. (17) did not include stability testing, for example during freeze–thaw cycles, which may limit the long-term reliability of their quantification results. In contrast to Wu *et al*. (17), Sari *et al*. (8) and Elliot *et al*. (1), we evaluated the selectivity of our method. This avoided any potential interferences and carry-over effects during analysis, especially because our calibration curve included very high and very low concentration points. The mean matrix effect for all species was not quite 12%, confirming that the developed method was influenced by matrix components to an acceptable extent by the criteria established.

The developed analytical method met all validation criteria, demonstrating superior sensitivity, precision and stability compared with data available in the literature. Minimal matrix effect, low LOD/LLOQ and comprehensive stability tests indicated greater reliability of this method for accurately measuring isoxazoline derivatives. The proposed method was successfully used to analyse the plasma of randomly selected cats and dogs from a small animal clinic for the presence of isoxazoline derivatives. This screening confirmed that the method worked well under real clinical conditions, and could be taken up for the detection and measurement of isoxazoline residues in plasma collected during routine veterinary visits. Such a screening test would reveal whether antiparasitic drugs had been used and give a veterinarian reliable information when there was no record of treatment. Therefore, the presented method can be useful not only in pharmacokinetic studies, but also for control purposes, especially in cases of suspected drug side effects or misuse, or of their undocumented administration. Given the growing use of isoxazoline-based treatments, developing a fast, cost-effective and reproducible screening method is essential for accurate monitoring and regulatory compliance.

Although the developed method met the criteria selected by the authors, the main limitation of the study was that the MS/MS protocol was designed to examine each isoxazoline compound separately. Another is that no isotopic internal standard was used in the study although such a standard would have been a better choice in tandem mass spectrometry to obtain more consistent results. In addition, the sample preparation method used in the study is a “dirty” technique that can shorten the life of the column. Despite these limitations, the proposed method is a reliable solution for determining isoxazoline derivatives in plasma. Further research is needed to address the identified shortcomings and achieve more satisfactory results.

## Conclusion

This is the first study to propose and validate a UPLC-MS/MS method for quantifying all isoxazoline derivatives in the blood plasma of laying hens, dogs, cats and humans. The results indicate that the developed method is replicable, precise, accurate, selective and sensitive. The main advantages of the method are its simplicity and effectiveness, as well as quick sample preparation, which decreases costs and facilitates rapid and precise determination of the analytes in plasma. Despite the existence of cleaner techniques of plasma purification than protein precipitation, the proposed method had a high recovery rate, and the matrix effect was small enough for the method to be validated with sufficient accuracy and precision. The method seems to be suitable for practical use, and was successfully applied to test for the isoxazoline derivatives fluralaner,sarolaner, lotilaner and afoxolaner in animals that had been orally administered isoxazolines.
